# Application of next-generation sequencing in the detection of transgenic crop

**DOI:** 10.3389/fgene.2024.1461115

**Published:** 2024-11-28

**Authors:** Shitian He, Yueyuan Fan, Shaomin Tao, Yanxia Zhang, Chuanlin Yin, Xiaoping Yu

**Affiliations:** Zhejiang Provincial Key Laboratory of Biometrology and Inspection and Quarantine, College of Life Sciences, China Jiliang University, Hangzhou, Zhejiang, China

**Keywords:** genetically modified crops, transgenic safety, genetically modified food detection, next-generation sequencing, high-throughput detection

## Abstract

With the rapid development of transgenic technology and the increasing prevalence of genetically modified (GMO) crops, incidents such as illegal importation, environmental contamination, and safety concerns associated with GMOs have risen significantly in recent years. Consequently, there is a growing demand for more advanced methods of GMO crop detection. Traditional molecular detection techniques, which rely on nucleic acids or proteins, are becoming less effective due to the increasing complexity of GMO crop genomes. In contrast, detection technologies based on second- and third-generation high-throughput sequencing offer promising solutions to these challenges. This review provides a comprehensive overview of the latest advancements in GMO crop detection technologies, categorizing and describing various approaches, and comparing their respective strengths and limitations. The article emphasizes the current state, benefits, challenges, and future prospects of high-throughput sequencing in GMO detection, aiming to guide further research and development in this field.

## 1 Introduction

Genetically modified (GM) crops are those in which exogenous genes have been introduced into the plant genome using genetic engineering techniques. These exogenous genes may originate from individuals within the same species, different species, or even entirely different organisms. The primary objectives of developing transgenic crops including improving crop quality, increasing yields, enhancing resistance to pests and diseases, and bolstering adaptability (Kamle and Ali, 2013). Transgenic breeding technology overcomes the limitation of traditional breeding methods by facilitating gene exchange between species and enabling the segregation of desirable and undesirable traits, thereby accelerating the breeding process (Ryding et al., 2001). Furthermore, transgenic technology offers a promising solution to global challenges such as resource scarcity, environmental degradation and food shortage by increasing crop yields, improving quality and enhancing resistance, making it a key technological pillar in modern agriculture. However, despite its efficiency, GM technology has been met with persistent global controversy, particularly concerning environmental and food safety (Bawa and Anilakumar, 2013). Environmental concerns primarily revolve around the potential for genetic drift and contamination (Kim et al., 2019), while Food safety concerns focus on the allergenicity of proteins expressed by transgenes, impacts on nutrient content, and the presence of natural toxins or antinutritional factors (Kumar et al., 2020). The insertion of new genetic material into the host genome may increase the levels of endogenous allergens, toxins or antinutrients, posing potential risk of sensitization or toxicity (Delaney et al., 2018).

To ensure the safe and sustainable development of GM crops and to meet consumer demands for transparency and choice, countries worldwide have established comprehensive GMO safety monitoring and evaluation system (Li, 2017). Many regions have implemented mandatory labeling systems for GM products, with varying degrees of stringency. For instance, Japan and South Korea require labeling for products containing more than 5% or 3% GM ingredients, respectively, while the European Union mandates labeling for products 0.9%. Since 2001, China has introduced stringent labeling regulations for agricultural GMOs, adopting a qualitative labeling system with stricter controls. Despite these measures, incidents involving the illegal importation, contamination, and safety breaches of GM products have increased, underscoring the need for enhanced testing and supervision. As the cultivation and international trade of GM crops expand, the development of simple, rapid, and effective on-site testing methods is crucial for ensuring compliance with safety standards and regulatory requirements. Rapid testing of GM crops is essential not only for verifying the authenticity of GM crops but also for safeguarding consumers’ rights to know and choose. Additionally, GM crop testing plays a vital role in monitoring the distribution and cultivation of GM crops, preventing unauthorized products from entering the market, and maintaining fair competition in agricultural production. Moreover, such testing is crucial for assessing the environmental impacts of GM crops, identifying potential risks, and protecting ecosystem stability and health. Therefore, the detection of genetically modified crops is integral to ensuring food and environmental safety, contributing significantly to sustainable agricultural development and human health.

**FIGURE 1:**
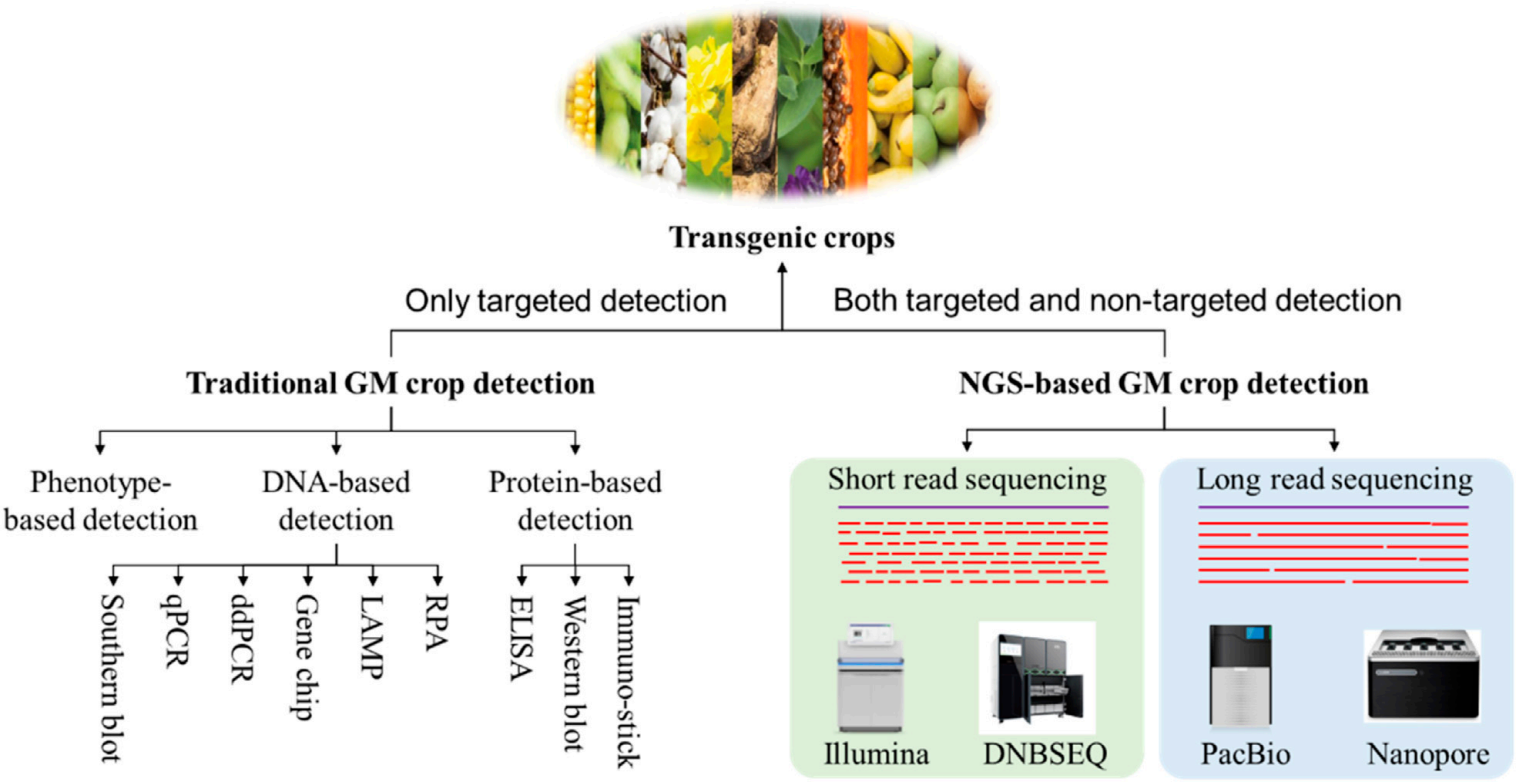
A schematic view of detection methods for transgenic crops.

Currently, the primary technologies employed for the detection of transgenic crops encompass phenotype-based detection, nucleic acid-based detection, protein-based detection and high-throughput sequencing-based detection methods (Figure 1; Debode et al., 2019). Of these, nucleic acid- and protein-based detection technologies are the most established and have traditionally served as the cornerstone of GM crop detection. This paper offers a concise review of the research advancements and practical applications of these various detection technologies. It critically examines the strengths and limitations of each approach within specific application contexts and seeks to outline the future trajectory of GM crop detection methodologies. The objective is to provide informed guidance for the ongoing development of effective GMO safety regulation.

## 2 Traditional GM crop detection methods

### 2.1 Phenotype-based GM crop detection techniques

Phenotype-based detection, also known as bioassay, is a method for distinguishing between GM and non-GM crops by observing specific transgenic traits in the samples (Kamle et al., 2017). This technique, while not requiring extensive equipment, is primarily used for the initial identification of viable transgenic crops. However, it is time-consuming and does not provide detailed information on the specific transgene sequence or its location. Phenotypic detection is commonly applied to identify traits such as herbicide resistance and insect resistance in crops (Goggin et al., 2015), with herbicide resistance bioassay technology being utilized in soybean, corn, cotton, and oilseed rape (Ladics et al., 2015). Despite its usefulness in confirming successful transgenic breeding, this method has significant limitations in precision and scope.

### 2.2 Nucleic acid-based GM crop detection techniques

Nucleic acid-based detection technologies, particularly polymerase chain reaction (PCR) methods, are widely employed for identifying specific transgenic DNA sequences in crops. These include techniques such as DNA blot (Southern blot), real-time fluorescence quantitative PCR (qPCR), droplet digital PCR (ddPCR), Gene chips, loop-mediated isothermal amplification (LAMP) and recombinase polymerase amplification (RPA) (Table 1). DNA blotting, a method that involves the hybridization of probes to DNA sample without amplifying of the sample DNA (Glenn and Andreou, 2013), is characterized by relatively low sensitivity (Green and Sambrook, 2021). Despite this limitation, Al-Baraa Akram demonstrated the feasibility, specificity, and reproducibility of DNA blotting in the detection of transgenic *Arabidopsis thaliana*, as well as in various other transgenic and non-transgenic cell lines (Akram et al., 2022). In contrast, real-time fluorescence quantitative PCR is a highly sensitive and specific assay that an assay technique that use fluorescent probes to monitor PCR product formation in real time. This method offers significant advantage in accuracy and speed, making it an indispensable tool for the quantitative detection of the nucleic acid copy number in transgenic samples (Broeders et al., 2014). Additionally, qPCR can simultaneously detect multiple transgenic crops, thereby enhancing both efficiency and accuracy, and is widely regarded as the preferred method for assessing transgenic content in food and feed (Cottenet et al., 2019; Xiong et al., 2017). However, the technique’s high costs, the complexity of its experimental procedures, and the need for precision instruments, coupled with its susceptibility to contamination and interference, necessitate strict control of experimental conditions. Microdroplet digital PCR further refines nucleic acid detection by partitioning the PCR reaction into water-in-oil microdroplets, each functioning as an independent reaction unit. This portioning significantly reduces matrix effects, thereby enhancing the sensitivity and accuracy of the assay (Moser et al., 2014). Studies have shown that microdroplet digital PCR outperforms both microarray digital PCR and fluorescence quantitative PCR in terms of quantitative sensitivity and accuracy (Morisset et al., 2013; Wei et al., 2022). Collier et al. (2017) effectively utilized this technique to accurately quantify transgene copy number in various transgenic crops, including rice, citrus, potato, maize, tomato and wheat. Unlike real-time qPCR, microdroplet digital PCR does not require a standard curve and surpassed qPCR in linear range, detection limit and quantification thresholds, Specifically, ddPCR can reliably detect as few as 1–5 copies of the target sequence per reaction, highlighting its superior sensitivity and accuracy for low-abundance target quantification (Burns et al., 2010). Gene chip, also known as DNA microarray, biochip or DNA microarray, is a high-throughput, high-efficiency, and highly automated method (Chen et al., 2019; Wang et al., 2021). In the context of transgenic crops detection, gene chip exhibit superior efficiency and accuracy, particularly in the analysis of composite samples or multi-sample detections (Hardinge et al., 2018). However, the widespread application of gene chips for transgene detection is limited by the high costs associated with chip preparation and the necessary equipment, indicating a need for further development to reduce costs and increase accessibility. Loop-mediated isothermal amplification (LAMP) is another technique used in the detection of transgenic crops. LAMP employs specifically designed primers to amplify target transgene sequences, with results observable either through fluorescence under ultraviolet light or directly by the naked eye via the presence of a white precipitate, thereby eliminating the need for electrophoresis. Compared to conventional PCR, LAMP offers superior specificity and sensitivity and reduces detection time (Hardinge et al., 2018; Wu et al., 2020). Recombinase polymerase amplification (RPA), a novel DNA isothermal amplification technique, has been hailed as a revolutionary advancement in DNA diagnostics (Xu et al., 2014b). RPA involves the formation of a complex between recombinant enzyme and primers, which then locates homologous sequences on double-stranded DNA, initiating strand exchange and DNA synthesis to amplify of the target region (Iftode et al., 1999; Xu et al., 2014a). This entire process can be completed within 10–20 min, significantly reducing detection time.

**TABLE 1:** Comparing nucleic acid-based methods for genetically modified crop detection.

Detection methods	Advantages	Disadvantages
DNA blotting	Easier to operate, less efficient, less expensive	Low accuracy, no quantitative detection
Real-time fluorescence quantitative PCR	Sensitive and simple operation, quantitative detection, real-time processing	Consumables are more expensive, less accurate, less efficient, need to draw calibration curves
Microtitre Digital PCR	Highly sensitive, highly quantitative, highly accurate, no need to draw calibration curves	Higher cost, limited throughput, cumbersome operation
Gene chip method	High throughput, high efficiency, high accuracy	High chip preparation costs and expensive instrumentation
Loop-mediated isothermal amplification	Portable, low equipment requirements, rapid detection, no thermal cycling process required	Low accuracy, no quantitative detection

### 2.3 Protein-based detection techniques for genetically modified crops

Protein-based detection techniques, including enzyme-linked immunosorbent assay (ELISA), Western blot, and immunochromatographic test strip method, are also widely used in GM crop dections (Table 2). ELISA, which operates on the immunological principle of antigen-antibody specificity combined with enzyme-substrate reactions, is particularly effective for detecting target proteins containing exogenous genes in GM crops. This method has gained widespread application in the identification and testing of GM crops (Gampala et al., 2019; Zhang et al., 2016). However, despite its convenience and efficiency, ELISA is primarily suited for raw material products, and its application in detecting processed foods is more challenging. Western blotting is another protein-based technique that detects the expression of specific proteins in cell tissues by using antibodies to bind these proteins. While effective, this method, like ELISA, has inherent limitations and is likely to be supplanted by more advanced and high-throughput quantifiable techniques in the future (Kurien and Scofield, 2006; Rong et al., 2016). Immunochromatographic test strip technology, developed in the early 1980s, has matured into a reliable method, often employed at customs ports and other entry points for the rapid initial screening of GM crops. This technique combines the strengths of chromatography and immunoassay, and is frequently used in conjunction with other technologies to detect genetically modified products (Ngom et al., 2010). It offers high specificity and stability (Wang et al., 2011). The advantages of immunostrip technology include its simplicity, rapid detection speed, low cost, and the fact that it does not require highly trained personnel for operation. However, like other protein-based detection methods, it has limitations, including a higher likelihood of false positives compared to alternative detection methods (Mazzara et al., 2013).

**TABLE 2:** Comparing protein-based methods for genetically modified crop detection.

Detection methods	Advantages	Disadvantages	
ELISA	Convenient, efficient, wider application, higher specificity	Genetically modified protein crops are difficult to detect and are not applicable to the detection of processed products with denatured proteins	For feedstock products, low throughput, non-quantifiable
Western blot	Easy to operate, relatively high sensitivity		Non-quantifiable, limitations in detection
Immunochromatographic test strip	Wide range of applications, low cost, easy to operate		High false positives, low accuracy, only for primary screening

## 3 Detection methods of transgenic crops based on high-throughput sequencing technology

### 3.1 High-throughput sequencing technology and its advantages in GM crop detection

High-Throughput Sequencing (HTS), also referred to as Next-Generation Sequencing (NGS), is a powerful technology that enables the rapid, accurately, and cost-effective determination of DNA or RNA sequences. Compared with the traditional Sanger sequencing, HTS offer a significantly higher capacity, allowing for the simultaneous processing of thousands to millions of DNA fragments, thereby generation substantial amounts of sequencing data in a considerable shorter timeframe. HTS is generally classified into two primary categories: second-generation and third-generation sequencing technologies. Second-generation HTS technologies including platforms such as Illumina, 454, SOLiD, and DNBSEQ, while third-generation sequencing technologies encompass platforms like PacBio, Oxford Nanopore (Table 3). Second-generation HTS is characterized by its high accuracy, throughput, and sensitivity, as well as its relatively low operating cost. However, it is limited by shorter sequencing read length, typically around 150 base pairs, and requires bulky, expensive equipment, leading to high platform construction costs and extended experimental cycle. In contrast, third-generation technologies such as PacBio, offer longer read length, PCR-free amplification, single-molecule sequencing, and the ability to detect DNA modifications, making them particularly useful for addressing genomic repetitive sequences and structural variants. However, these advantages come with trade-offs, including a higher error rate, increased cost, and more complex data processing requirement. While second-generation HTS generally offers higher accuracy and lower costs compared with third-generation sequencing, it faces challenges in detecting longer transgenic sequences due to its shorter read lengths. Additionally, the underlying principles of second-generation methods can sometimes result in false-positive or false-negative outcomes, and the technology may struggle with complex sample handling and data analysis. On the other hand, third-generation Oxford Nanopore sequencing, a newer single-molecule real-time sequencing technology, has matured in recent years. Unlike earlier technologies that rely on chemical signals, Nanopore sequencing detects bases by measuring the changes in potential difference as nucleic acid molecules pass through a nanopore (Branton et al., 2008). This allows for direct sequencing of DNA or RNA sequences without the need for complex pre-processing steps such as PCR amplification or library construction. Nanopore sequencing offers several unique advantages, including faster sequencing speeds, longer read lengths, real-time data analysis, and a portable, easy-to-use platform with lower overall costs (Collier et al., 2017), making it particularly suitable for detecting complex gene recombination events and genome structural variants. Despite these advantages, Nanopore sequencing is not without its challenges. The technology’s higher error rate in individual read sequences necessitates multiple sequencing runs and error correction to achieve the desired accuracy. Furthermore, the high cost of equipment and consumables associated with Nanopore sequencing may limit its applicability for large-scale transgene detection. In summary, both third-generation nanopore sequencing technology and second-generation HTS have distinct advantages and limitations, and the choice of the appropriate detection method should be guided by the specific requirements of the experimental context.

**TABLE 3:** Comparing genetically modified crop detection methods based on different sequencing technologies.

Detection methods	Advantages	Disadvantages
Illumina, DNBSEQ	Highest accuracy and lowest cost for single base sequencing	Short read lengths, complex processes, long sequencing cycles
PacBio	Long read length, no PCR amplification, single molecule sequencing	Higher sequencing error rate, higher cost of single base sequencing, complex process, long sequencing lead time
Nanopore	Longest read length, no PCR amplification required, single molecule sequencing, can analyze while sequencing, simple and low cost equipment, simple process, short sequencing cycle time	Highest sequencing error rate, highest single base sequencing cost

Compared to traditional detection methods, GM crop detection utilizing high-throughput sequencing (HTS) technology offers significant inherent advantages in both detection and regulatory processes, effectively addressing many of the limitations associated with conventional approaches (Debode et al., 2019). Firstly, HTS exhibits exceptionally high sensitivity, capable of detecting DNA sequences at very low concentrations. This heightened sensitivity allows for the reliable identification of transgenic sequences, even when present in minimal amounts within a sample. Secondly, HTS provides high specificity, enabling precise recognition and differentiation of DNA sequences from various host sources. This accuracy ensures the correct identification of specific transgene sequences in GM crops, thereby minimizing the risk of misclassification and detection errors. Additionally, HTS technology boasts the capacity to process large volumes of samples simultaneously, significantly enhancing the efficiency and productivity of large-scale GM crop testing. This high-throughput capability allows for the rapid completion of extensive testing tasks, which is crucial in regulatory environments where timely results are essential. Furthermore, HTS is an inherently unbiased detection method, not constrained by prior knowledge of the detection target. It is capable of comprehensively analyzing all DNA sequences within a sample, including transgenic sequences, as well as other potential hybridization events or mutations (Abel and Duncavage, 2013). Finally, the data generated by HTS is fully digitized, enabling efficient archiving and traceability, which is essential for quality control and the validation of results. This traceability enhances the credibility and reliability of the detection outcomes, making HTS a superior tool for GM crop detection and regulation. In summary, HTS offers enhanced sensitivity, specificity, throughput, and traceability compared to traditional detection methods, providing a more effective and reliable approach for the detection and regulation of genetically modified crops.

### 3.2 Research progress on algorithms and processes for analyzing transgenic crop data based on high-throughput sequencing technology

In the detection of GM crops using high-throughput sequencing (HTS) technology, the accuracy of crop identification is heavily dependent on the data analysis process and the detection and identification algorithms employed. The development of these algorithms is an area of active research, with notable advancements reflected in software such as TDNAscan (Sun et al., 2019) and T-LOC (Li et al., 2022), both of which represent mature solutions in this domain. TDNAscan, developed in Python, is specifically designed to identify T-DNAs that are either integrated or truncated within GM crops. The core algorithmic process involves comparing sequenced data with T-DNA sequences and reference genome sequences. The software filters sequences that match either the T-DNA or the reference genome, categorizing and combining them based on the CIGAR values of the matched files to pinpoint T-DNA insertion sites (Sun et al., 2019). While TDNAscan effectively detects truncated T-DNA insertions, its validation has been limited to *A. thaliana*, and the results it generates are not presented graphically, which may limit its utility in broader applications. Conversely, T-LOC is a more versatile tool, developed using Python and R, designed to manage the detection and identification of transgenic plants exhibiting diverse T-DNA patterns. In a study analyzing genome sequencing data from 48 transgenic rice plants, T-LOC successfully identified 75 complete T-DNA integration sites, demonstrating its capability in handling diverse T-DNA configurations. The primary algorithmic approach of T-LOC involves initially comparing sequencing data with both the reference genome and vector sequences. Unmatched portions of the sequence are then re-compared with sequences from the plant’s reference genome and the vector, allowing for certain mismatches and insertions. The software subsequently filters out soft-clipped sequences that are fully present in the reference genome and those without T-DNA integration. Finally, T-LOC generates four types of outputs for further data analysis. Additionally, the software offers graphical visualization of the final insertion sites, enabling a more intuitive understanding of genome sequences, gene locations, mutation distributions, and other critical genomic information. This graphical presentation of data enhances the interpretability of results, making T-LOC a powerful tool for transgenic plant research and contributing to a more comprehensive understanding of genomic modifications in GM crops.

In addition to the aforementioned software tools, several other algorithms are instrumental in the identification of insertion sites and the detection of mutations in transgenic crops, including VariationHunter, BreakDancer, FNBtools, and ITIS. FNBtools, for instance, is designed to identify pure lesions within deletion mutation populations (Sun et al., 2019), while ITIS is specifically tailored for identifying insertion sites in simulated legume genomes using next-generation sequencing (NGS) data (Jiang et al., 2015). VariationHunter is a prominent algorithm used for detecting structural variants in high-throughput sequencing data by comparing the sequencing data against a reference genome. It identifies structural variants such as insertions, deletions, inversions, and duplications, employing a combinatorial algorithm to enhance the accuracy of detection. Notably, VariationHunter is adaptable to datasets with varying sequencing depths and coverage, making it widely applicable in genomic research and the study of human diseases (Hormozdiari et al., 2010). Similarly, BreakDancer is another well-regarded algorithm for structural variant detection. It operates by analyzing fragment alignment patterns within sequencing data to identify abnormalities, thereby detecting various structural variants, including insertions, deletions, inversions, duplications, and transposons. BreakDancer is characterized by its high resolution and sensitivity and is versatile enough to be adapted to sequencing data with different depths and fragment lengths. Due to its robust performance, BreakDancer is extensively used in the study of genomic structural variants (Chen et al., 2009).

### 3.3 Difficulties faced in the application of high-throughput sequencing technology in the detection of genetically modified crops

Despite the inherent advantages of high-throughput sequencing (HTS) technology over traditional methods in GM crop detection, it continues to face several significant challenges related to sequencing, data analysis, cost, and standardization. Firstly, the implementation of HTS for GM crop detection is associated with substantial financial and logistical demands. The high cost of HTS equipment, reagents, and maintenance, coupled with the need for a well-equipped laboratory environment and highly skilled technicians for both operation and data analysis, renders this technology prohibitively expensive for many small and medium-sized laboratories or testing facilities with limited resources. Secondly, HTS generates vast amounts of data, and the subsequent analysis process is both complex and time-consuming, necessitating powerful computational resources and specialized bioinformatics expertise. The challenges are further compounded when analyzing complex samples containing multiple transgenes or mixed genetic material, as the presence of noise and background signals—especially when detecting low-abundance transgenic fragments—can significantly complicate data interpretation. Moreover, there is a notable gap in time and efficiency between HTS and traditional detection methods. Although HTS has the potential to rapidly generate large volumes of data, the entire process, from sample preparation through sequencing to data analysis, remains time-intensive. This limitation is particularly problematic when quick detection results are required, as the current HTS protocols may not be sufficiently efficient. Lastly, the lack of global standardization in HTS-based GM crop testing methods presents a major challenge. Different laboratories may employ varying sequencing platforms and data analysis procedures, leading to inconsistencies and reduced comparability of results. The absence of uniform international standards and norms has consequently limited the acceptance of HTS-based test results at regulatory and legal levels. To overcome these challenges, technological advancements are necessary to reduce costs, enhance automation, and standardize data analysis processes. Establishing internationally recognized standards and norms will be crucial in ensuring the broader adoption and reliability of HTS in GM crop detection. Additionally, policy and regulatory reforms will be essential to facilitate the widespread application of HTS technology in this field.

## 4 Conclusion and perspectives

The continuous advancement of transgenic engineering technology, coupled with the rapid proliferation of transgenic crop varieties and quantities, has significantly complicated the detection of transgenic components, especially in cases where foodstuffs undergo extensive processing that may lead to partial or complete degradation of these components (Grohmann et al., 2019). Traditional GM crop detection techniques—whether based on phenotype, nucleic acid, or protein analysis—are heavily dependent on existing research foundations and require detailed knowledge of the genetic background of the crops in question. Consequently, these methods are limited to the targeted detection of known GM crops and are ineffective for identifying new GM crops with unknown genetic backgrounds. In recent years, the challenges associated with the illegal importation of GM products, contamination, and safety incidents have escalated, rendering traditional GM detection methods increasingly inadequate. As a result, there is a growing need for detection methodologies that can effectively identify GM products with unknown genetic sequences and insertion sites. High-throughput sequencing (HTS) technology has emerged as a particularly vital tool in this context, offering the capability to detect GM products regardless of prior knowledge of their genetic makeup (Table 4).

**TABLE 4:** Summary and comparison of various detection methods.

Classify	Advantages	Disadvantages	
Phenotype-based assays	Direct, easy and fast	Unable to locate the location of the transgene, unable to detect unknown transgene sequences	Heavily relies on the existing research base and must have sufficiently clear information on the genetic background of the GM crop, and therefore can only perform targeted testing on known GM crops, and cannot be performed on new GM crops with unknown backgrounds, and producing a certain number of false positives
Nucleic acid-based assays	Widely used, easy and quick to operate	Unable to detect transgenic products of unknown sequence, low throughput	
Protein-based assays	Widely used and easy to operate	Unable to detect genetically modified products of unknown sequence, not suitable for detection of processed products, and difficult to prepare antibodies	
High-throughput sequencing-based assays	High sensitivity, high accuracy, high throughput, no bias, data traceability	Unable to achieve rapid detection, high cost and complex data analysis	

Compared to the traditional nucleic acid- or protein-based methods currently prevalent in GM crop detection, high-throughput sequencing (HTS) technology offers substantial advantages. These include superior sensitivity, specificity, and high-throughput capacity, as well as the ability to detect GM crops without requiring prior knowledge of their genetic background or reliance on existing research foundations. However, despite these benefits, the application of HTS in GM crop detection remains largely confined to the research and demonstration stage. This is primarily due to its high costs, complex data analysis requirements, and the lack of standardization across platforms and protocols, which currently limits its broader adoption and relegates it to a supplementary role alongside traditional methods. Future research and development efforts should focus on several key areas to facilitate the wider adoption of HTS technology. First, reducing costs through technological innovation and economies of scale will be essential to make HTS accessible to small and medium-sized laboratories and testing facilities. Second, optimizing data processing algorithms and simplifying the analysis process will improve efficiency, ensuring the accuracy and consistency of results. Third, there is a pressing need to advance global standardization efforts, establishing uniform testing standards and specifications to enh ance the comparability and recognition of HTS results across different laboratories. Finally, the development of regulatory and ethical frameworks for HTS application should be accelerated to ensure that testing processes and outcomes comply with the legal requirements of each country. By addressing these challenges, HTS technology has the potential to become a more reliable and effective solution for transgenic crop detection, ultimately contributing significantly to the biosafety and ecological protection of transgenic crops.

## References

[B1] Abel H. J. Duncavage E. J. (2013). Detection of structural DNA variation from next generation sequencing data: a review of informatic approaches. Cancer Genet. 206 (12), 432–440. 10.1016/j.cancergen.2013.11.002 24405614 PMC4441822

[B2] Akram A.-B. McCann G. Sgamma T. (2022). Identification transgenic DNA of transformed Arabidopsis using PCR and southern blot. Adv. Biosci. Biotechnol. 13 (3), 134–144. 10.4236/abb.2022.133006

[B3] Bawa A. Anilakumar K. (2013). Genetically modified foods: safety, risks and public concerns—a review. J. food Sci. Technol. 50 (6), 1035–1046. 10.1007/s13197-012-0899-1 24426015 PMC3791249

[B4] Branton D. Deamer D. W. Marziali A. Bayley H. Benner S. A. Butler T. (2008). The potential and challenges of nanopore sequencing. Nat. Biotechnol. 26 (10), 1146–1153. 10.1038/nbt.1495 18846088 PMC2683588

[B5] Broeders S. Huber I. Grohmann L. Berben G. Taverniers I. Mazzara M. (2014). Guidelines for validation of qualitative real-time PCR methods. Trends food Sci. and Technol. 37 (2), 115–126. 10.1016/j.tifs.2014.03.008

[B6] Burns M. J. Burrell A. M. Foy C. A. (2010). The applicability of digital PCR for the assessment of detection limits in GMO analysis. Eur. Food Res. Technol. 231, 353–362. 10.1007/s00217-010-1277-8

[B7] Chen B. Wang S. Hong B. Zhao Y. (2019). “Gene chips for food quality evaluation,” in Evaluation technologies for food quality (Elsevier). 10.1016/B978-0-12-814217-2.00024-X

[B8] Chen K. Wallis J. W. McLellan M. D. Larson D. E. Kalicki J. M. Pohl C. S. (2009). BreakDancer: an algorithm for high-resolution mapping of genomic structural variation. Nat. methods 6 (9), 677–681. 10.1038/nmeth.1363 19668202 PMC3661775

[B9] Collier R. Dasgupta K. Xing Y. P. Hernandez B. T. Shao M. Rohozinski D. (2017). Accurate measurement of transgene copy number in crop plants using droplet digital PCR. Plant J. 90 (5), 1014–1025. 10.1111/tpj.13517 28231382

[B10] Cottenet G. Blancpain C. Sonnard V. Chuah P. F. (2019). Two FAST multiplex real-time PCR reactions to assess the presence of genetically modified organisms in food. Food Chem. 274, 760–765. 10.1016/j.foodchem.2018.09.050 30373005

[B11] Debode F. Hulin J. Charloteaux B. Coppieters W. Hanikenne M. Karim L. (2019). Detection and identification of transgenic events by next generation sequencing combined with enrichment technologies. Sci. Rep. 9 (1), 15595. 10.1038/s41598-019-51668-x 31666537 PMC6821802

[B12] Delaney B. Goodman R. E. Ladics G. S. (2018). Food and feed safety of genetically engineered food crops. Toxicol. Sci. 162 (2), 361–371. 10.1093/toxsci/kfx249 29211881

[B13] Gampala S. S. Wulfkuhle B. Richey K. A. (2019). Detection of transgenic proteins by immunoassays. Transgenic Plants Methods Protoc. 1864, 411–417. 10.1007/978-1-4939-8778-8_25 30415349

[B14] Glenn G. Andreou L.-V. (2013). “Analysis of DNA by southern blotting,” in Methods in enzymology (Elsevier). 10.1016/B978-0-12-418687-3.00005-7 24011036

[B15] Goggin F. L. Lorence A. Topp C. N. (2015). Applying high-throughput phenotyping to plant–insect interactions: picturing more resistant crops. Curr. Opin. Insect Sci. 9, 69–76. 10.1016/j.cois.2015.03.002 32846711

[B16] Green M. R. Sambrook J. (2021). Southern blotting. Cold Spring Harb. Protoc. 2021 (7), pdb.prot100487. pdb. prot100487. 10.1101/pdb.prot100487 34210769

[B17] Grohmann L. Keilwagen J. Duensing N. Dagand E. Hartung F. Wilhelm R. (2019). Detection and identification of genome editing in plants: challenges and opportunities. Front. Plant Sci. 10, 236. 10.3389/fpls.2019.00236 30930911 PMC6423494

[B18] Hardinge P. Kiddle G. Tisi L. Murray J. A. (2018). Optimised LAMP allows single copy detection of 35Sp and NOSt in transgenic maize using Bioluminescent Assay in Real Time (BART). Sci. Rep. 8 (1), 17590. 10.1038/s41598-018-36207-4 30514874 PMC6279926

[B19] Hormozdiari F. Hajirasouliha I. Dao P. Hach F. Yorukoglu D. Alkan C. (2010). Next-generation VariationHunter: combinatorial algorithms for transposon insertion discovery. Bioinformatics 26 (12), i350–i357. 10.1093/bioinformatics/btq216 20529927 PMC2881400

[B20] Iftode C. Daniely Y. Borowiec J. A. (1999). Replication protein A (RPA): the eukaryotic SSB. Crit. Rev. Biochem. Mol. Biol. 34 (3), 141–180. 10.1080/10409239991209255 10473346

[B21] Jiang C. Chen C. Huang Z. Liu R. Verdier J. (2015). ITIS, a bioinformatics tool for accurate identification of transposon insertion sites using next-generation sequencing data. BMC Bioinforma. 16, 72–78. 10.1186/s12859-015-0507-2 PMC4351942 25887332

[B22] Kamle M. Kumar P. Patra J. K. Bajpai V. K. (2017). Current perspectives on genetically modified crops and detection methods. 3 Biotech. 7, 219–315. 10.1007/s13205-017-0809-3 PMC5495694 28674844

[B23] Kamle S. Ali S. (2013). Genetically modified crops: detection strategies and biosafety issues. Gene 522 (2), 123–132. 10.1016/j.gene.2013.03.107 23566850

[B24] Kim H. J. Kim D. Y. Moon Y. S. Pack I. S. Park K. W. Chung Y. S. (2019). Gene flow from herbicide resistant transgenic soybean to conventional soybean and wild soybean. Appl. Biol. Chem. 62, 54–58. 10.1186/s13765-019-0461-1

[B25] Kumar K. Gambhir G. Dass A. Tripathi A. K. Singh A. Jha A. K. (2020). Genetically modified crops: current status and future prospects. Planta 251 (4), 91. 10.1007/s00425-020-03372-8 32236850

[B26] Kurien B. T. Scofield R. H. (2006). Western blotting. Methods 38 (4), 283–293. 10.1016/j.ymeth.2005.11.007 16483794

[B27] Ladics G. S. Bartholomaeus A. Bregitzer P. Doerrer N. G. Gray A. Holzhauser T. (2015). Genetic basis and detection of unintended effects in genetically modified crop plants. Transgenic Res. 24, 587–603. 10.1007/s11248-015-9867-7 25716164 PMC4504983

[B28] Li F. W. (2017). Development of molecular detection methods for genetically modified crops and creation of AgGlpF gene-transformed salt-tolerant soybean materials.

[B29] Li S. Wang C. You C. Zhou X. Zhou H. (2022). T-LOC: a comprehensive tool to localize and characterize T-DNA integration sites. Plant Physiol. 190 (3), 1628–1639. 10.1093/plphys/kiac225 35640125 PMC9614469

[B30] Mazzara M. Paoletti C. Corbisier P. Grazioli E. Larcher S. Berben G. (2013). Kernel lot distribution assessment (KeLDA): a comparative study of protein and DNA-based detection methods for GMO testing. Food Anal. Methods 6, 210–220. 10.1007/s12161-012-9407-5

[B31] Morisset D. Štebih D. Milavec M. Gruden K. Žel J. (2013). Quantitative analysis of food and feed samples with droplet digital PCR. PloS one 8 (5), e62583. 10.1371/journal.pone.0062583 23658750 PMC3642186

[B32] Moser D. A. Braga L. Raso A. Zacchigna S. Giacca M. Simon P. (2014). Transgene detection by digital droplet PCR. PLoS One 9 (11), e111781. 10.1371/journal.pone.0111781 25375130 PMC4222945

[B33] Ngom B. Guo Y. Wang X. Bi D. (2010). Development and application of lateral flow test strip technology for detection of infectious agents and chemical contaminants: a review. Anal. Bioanal. Chem. 397, 1113–1135. 10.1007/s00216-010-3661-4 20422164

[B34] Rong R.-j. Wu P.-c. Lan J.-p. Wei H.-f. Jian W. Hao C. (2016). Western blot detection of PMI protein in transgenic rice. J. Integr. Agric. 15 (4), 726–734. 10.1016/S2095-3119(15)61053-X

[B35] Ryding A. Sharp M. Mullins J. (2001). Conditional transgenic technologies. J. Endocrinol. 171 (1), 1–14. 10.1677/joe.0.1710001 11572785

[B36] Sun L. Ge Y. Sparks J. A. Robinson Z. T. Cheng X. Wen J. (2019). TDNAscan: a software to identify complete and truncated T-DNA insertions. Front. Genet. 10, 685. 10.3389/fgene.2019.00685 31428129 PMC6690219

[B37] Wang L. Luo Y. Zhou Q. Lai P. Zhang X. Bai Y. (2011). Application of nucleic acid strips in the detection of transgenic EPSPS crops. Lett. Biotechnol. 22 (2), 238–242. 10.3969/j.issn.1009-0002.2011.02.021

[B38] Wang Y. Zhao Y. Bollas A. Wang Y. Au K. F. (2021). Nanopore sequencing technology, bioinformatics and applications. Nat. Biotechnol. 39 (11), 1348–1365. 10.1038/S41587-021-01108-X 34750572 PMC8988251

[B39] Wei S.-X. Chen H.-X. Hu S. Zhao Y.-X. Shi H.-X. Wang Z. (2022). Application of duplex droplet digital PCR detection of miR-888 and miR-891a in semen identification. Fa yi xue za Zhi. 38 (6), 719–725. 10.12116/j.issn.1004-5619.2021.510802 36914387

[B40] Wu H. Zhang X. Wu B. Qian C. Zhang F. Wang L. (2020). Rapid on-site detection of genetically modified soybean products by real-time loop-mediated isothermal amplification coupled with a designed portable amplifier. Food Chem. 323, 126819. 10.1016/j.foodchem.2020.126819 32334306

[B41] Xiong J. Huang Q. Wu L. Cai D. Zhang Z. Huang X. (2017). Uncertainty in detecting event-specific fragments of genetically modified maize BT176 by quantitative real time PCR. J. Anhui Agric. Univ. 44 (1), 119–123. 10.13610/j.cnki.1672-352x.20170208.023

[B42] Xu C. Li L. Jin W. Wan Y. (2014a). Event-specific real-time RPA detection of transgenic rice kefeng 6. GMO Biosaf. Res. 5 (1). 10.5376/gmo.2014.05.0001

[B43] Xu C. Li L. Jin W. Wan Y. (2014b). Recombinase polymerase amplification (RPA) of CaMV-35S promoter and nos terminator for rapid detection of genetically modified crops. Int. J. Mol. Sci. 15 (10), 18197–18205. 10.3390/ijms151018197 25310647 PMC4227211

[B44] Zhang Y. Zhang W. Liu Y. Wang J. Wang G. Liu Y. (2016). Development of monoclonal antibody-based sensitive ELISA for the determination of Cry1Ie protein in transgenic plant. Anal. Bioanal. Chem. 408, 8231–8239. 10.1007/s00216-016-9938-5 27659816

